# From Vascular Dysfunction to Atherothrombosis: The Pivotal Role of Eicosanoids and Their Receptors in Platelet and Endothelial Imbalance: A Scoping Review

**DOI:** 10.3390/ijms27010162

**Published:** 2025-12-23

**Authors:** Giovanna Ritorto, Sara Ussia, Roberta Macrì, Maria Serra, Annamaria Tavernese, Carmen Altomare, Denise Maria Dardano, Chiara Idone, Ernesto Palma, Carolina Muscoli, Maurizio Volterrani, Francesco Barillà, Vincenzo Mollace, Rocco Mollace

**Affiliations:** 1 Pharmacology Laboratory, Institute of Research for Food Safety and Health (IRC-FSH), Department of Health Sciences, University “Magna Graecia” of Catanzaro, 88100 Catanzaro, Italy; giovanna.ritorto@studenti.unicz.it (G.R.); saraussia1598@gmail.com (S.U.); carmen.altomare@studenti.unicz.it (C.A.); denisemaria.dardano@studenti.unicz.it (D.M.D.);; 2 Laboratory of Mass Spectrometry and Proteomics, Department of Experimental and Clinical Medicine, University “Magna Graecia” of Catanzaro, 88100 Catanzaro, Italy; 3 Department of Medicine and Surgery, University Campus Bio-Medico of Rome, 00128 Rome, Italy; an.tavernese@gmail.com; 4 Veterinary Pharmacology Laboratory, Institute of Research for Food Safety and Health (IRC-FSH), Department of Health Sciences, University “Magna Graecia” of Catanzaro, 88100 Catanzaro, Italy; palma@unicz.it; 5 Cardiology Department, IRCCS San Raffaele Roma, Via di Valcannuta 250, 00166 Rome, Italy; maurizio.volterrani@sanraffaele.it; 6 Department of Experimental Medicine, University “Tor Vergata” of Rome, 00133 Rome, Italy; 7 Renato Dulbecco Institute, 88046 Lamezia Terme, Italy

**Keywords:** eicosanoids, eicosanoid receptors, atherothrombosis, endothelial dysfunction, platelet dysfunction, platelet aggregation, prostaglandins

## Abstract

Vascular endothelium balances antithrombotic and anti-inflammatory activity to control blood vessel tone under physiological conditions. However, endothelial dysfunction impairs these processes, causing a state that promotes clotting and inflammation. Eicosanoids are a major class of bioactive lipid mediators crucial for modulating endothelial and platelet function. Research has highlighted the roles of eicosanoids in vascular diseases, showing pro-inflammatory, prothrombotic, and protective activities. Specifically, prostaglandin E_2_ (PGE_2_) is crucial because of its major role in atherosclerosis development and progression, acting via EP receptors involved in forming, maintaining, and stabilizing atherosclerotic lesions, thereby making PGE_2_-EP signalling a specific target for treating cardiovascular diseases. This review will explore the evidence on eicosanoids and the role of their receptor modulation in platelet and vascular dysfunction in atherothrombosis. The studies included in this scoping review were retrieved from PubMed, Web of Science, Cochrane, and Scopus in accordance with the Preferred Reporting Items for Scoping Reviews and Meta-Analyses extension for Scoping Reviews (PRISMA-ScR) statement and the Population Intervention Comparison Outcome Population (PICO) framework. Eight clinical studies were found, which highlighted the crucial role of eicosanoids, like prostaglandins and their receptors, in endothelial and platelet dysfunction, and also how pharmacological mechanisms affect atherothrombosis. A new therapeutic approach for cardiovascular dysfunction is indicated by the recent findings, specifically against atherothrombosis, focusing on eicosanoids, their receptors, and processes like oxidative stress. Despite this evidence, there is a lack of comprehensive research results from scientific databases; therefore, further in vitro, in vivo, and clinical studies should be promoted to validate the preliminary results.

## 1. Introduction

The vascular endothelium, also referred to as “the natural blood vessel”, represents the continuous cellular surface of the cardiovascular system [1].

Endothelial cells (ECs) cover all vertebrate blood vessels, creating a large surface area, estimated at 3000–6000 m^2^ [2].

As a crucial anatomical and functional part of all human tissues and organs, the endothelium is the most widely distributed organ, with a mass similar to important organs like the kidney [3].

Specifically, the vascular endothelium is a system that shows dynamic adaptability, with changeable structural and functional properties due to stimulation from systemic and local sources [4]. Evidence indicates that ECs perform various location-specific tasks and show notable phenotypic differences throughout blood vessels [2,5].

The functionality of ECs is characterized by their versatility, and these cells are responsible for a variety of regulatory processes, which include permeability regulation, cell adhesion promotion, transport of fluids from the blood to the underlying cells and tissues, and control of vascular tone. Focusing on vascular relaxation/constriction, the leakage of solutes, fluids, hormones, and macromolecules promotes smooth muscle cell growth, angiogenesis, and vascular wall inflammation [5].

Indeed, ECs are able to respond to physical and chemical signals by synthesizing a wide range of factors that regulate these processes [2,5].

Under physiological conditions, the vascular endothelium exhibits an antithrombotic and anti-inflammatory phenotype and regulates vascular tone. It prevents thrombosis through various anticoagulant and antiaggregant molecular mechanisms and controls the expression of binding sites for anticoagulant and procoagulant factors on its surface [3,6].

In resistance arteries, ECs can control vascular tone, blood pressure, and local perfusion by releasing vasoactive factors. Nitric oxide (NO) and prostacyclin (PGI_2_) are major vasodilators, unlike the vasoconstrictors thromboxane A_2_ (TXA_2_) and endothelin-1 (ET-1) [7]. Endothelial nitric oxide synthase (eNOS) synthesizes NO, a vasodilator with a very short half-life, from L-arginine, particularly when platelets adhere to the intact arterial endothelium.

When released, NO promotes relaxation of the vascular smooth muscle by activating cyclic guanosine monophosphate (cGMP) and simultaneously exerts an antithrombotic effect by inhibiting the coagulation pathway and TXA_2_ receptor-mediated platelet aggregation [8].

Vasodilation and platelet activation are both inhibited by NO and prostaglandins (prostaglandin I (PGI) and prostaglandin E (PGE)), which are released by the endothelium to synergistically control anticoagulant function in the vasculature [9]. Alongside molecule secretion, various endothelial components contribute to the regulation of the physiological process. The endothelial glycochalice, a glycoprotein and proteoglycan matrix, forms a layer between ECs and blood. This layer protects blood vessels, maintaining their structure via permeability, signalling, and erythrocyte cell wall interactions [8].

Heparan sulphates are glycosaminoglycans found on endothelium surfaces and surrounding tissues; they can show anticoagulant activity when interacting with antithrombin. Specifically, antithrombin is a major inhibitor of both thrombin and factor Xa [8]. Protein C is triggered by the thrombin–thrombomodulin interaction, which deactivates factors Va and VIIIa. Additionally, the tissue factor pathway inhibitor (TFPI), present on ECs, stops the extrinsic coagulation pathway by inhibiting the action of factor VIIa/tissue complex and factor Xa [10].

The previously described properties of ECs are characteristic of a healthy endothelium; however, endothelial dysfunction is a pathological condition in which these functions are often compromised or changed into a prothrombotic state because of cardiovascular risk factors, inflammatory mediators, or procoagulant signals [11].

Endothelial dysfunction involves a pathological alteration in ECs, causing them to change from a stable state to one with pro-adhesive, pro-inflammatory, and prothrombotic properties [12]. A range of factors, encompassing recognized cardiovascular risks, like high blood pressure, diabetes, and high cholesterol, plus changeable lifestyle habits, such as smoking and obesity, contribute to the multifaceted condition of endothelial dysfunction. Additionally, age-related endothelial changes, chronic inflammation, heightened oxidative stress, hemodynamic issues, and genetics also play a role in the development of endothelial dysfunction, highlighting its intricate and varied causes [12].

Endothelial NO homeostasis imbalance is the primary cause of this phenotypic shift. Compromised vasodilation and a pro-inflammatory vascular state can arise from reduced eNOS activity or increased NO inactivation by superoxide [6].

Endothelial dysfunction is critically linked to reactive oxygen species (ROS) and high oxidative stress. Endothelial impairment is mainly caused by an imbalance between excess ROS and antioxidant defences. Redox dysregulation is a key factor in the vascular injury seen in metabolic disorders and atherosclerosis [6].

Atherosclerosis is a long-term inflammatory condition that impacts arteries, causing significant illness and death globally, mostly through heart attacks and strokes, and also through peripheral arterial disease [13]. Elevated levels of low-density lipoproteins (LDLs), smoking, hypertension, and diabetes are a few cardiovascular risk factors that cause oxidative stress, leading to increased ROS [14]. The increase in endothelial permeability, as a consequence of endothelial dysfunction, results in the penetration of LDLs and accumulation in the subendothelial space, where they are oxidized by ROS (produced by dysfunctional endothelium), resulting in oxidized low-density lipoproteins (ox-LDLs) [3]. Oxidized LDLs cause damaged endothelium to attract inflammatory cytokines, adhesion molecules, and chemotactic signals [15]. This response causes leukocytes, particularly monocytes, to stick to the endothelium, move into the intima, and become macrophages. Scavenger receptors allow macrophages to take in ox-LDL, which then become foam cells, forming fatty streaks [16].

However, the pro-inflammatory state, which is typical of the endothelial status and chronic inflammation, triggers the proliferation or recruitment of smooth muscle cells (SMCs) from the media to the intima, leading to the synthesis of extracellular matrix (ECM) (collagen, proteoglycans), the build-up of connective tissue, and the formation of the fibrous capsule that covers the lipid core, thus causing atherosclerotic plaque formation [7]. Stable plaques have thick fibrous layers, little lipid, and are less likely to burst. Unstable plaques have thin layers, are lipid-rich, and are prone to rupture. Plaques can be destroyed by two main processes: the fibrous capsule can be blocked by metalloproteinases (MMPs) from macrophages, which degrade the extracellular matrix, weakening the capsule, or through endothelial erosion [17]. The fibrous cap’s mechanical strength and the hemodynamic stress disparity determine plaque vulnerability to rupture [10]. Plaque disruption boosts thrombotic events, exposing thrombogenic core components, like Tissue Factor (TF) and collagen, which causes rapid platelet and coagulation activation, leading to thrombus formation [10]. Arterial occlusive events are usually caused by atherosclerotic plaques, not intact walls. Because of this pathophysiological connection, the term atherothrombosis, highlighting the link between atherosclerosis and blood clot formation, is used for heart attacks, unstable angina, and strokes [18,19].

Eicosanoids, a large group of bioactive molecules discovered since the 1930s, are oxidative metabolites of arachidonic acid (AA), which is a 20-carbon polyunsaturated fatty acid usually esterified in membrane phospholipids [20].

The term “eicosanoid” is rooted in the Greek word “eikosi”, signifying twenty: it was first used to describe lipids that were structurally similar to AA, which has twenty carbon atoms [21]. The generation of eicosanoids depends on cytosolic phospholipase A2 (cPLA2), which extracts AA from membrane phospholipids due to stimulation by factors like mechanical injury, cytokines, or growth signals; prostaglandin synthesis mainly occurs at the endoplasmic reticulum and nuclear membranes. The regulation of this crucial step is controlled by the type IV cPLA2 isoform, which is activated by calcium ions [22]. Subsequently, AA is delivered to cyclooxygenase 1 (COX-1) or cyclooxygenase 2 (COX-2), which is also called prostaglandin H synthase (PGHS) [22].

Prostaglandin G2 (PGG_2_), a short-lived intermediate, is first produced by the cyclooxygenase (COX) reaction and then quickly converted to prostaglandin H2 (PGH_2_), involving two COX isoforms: COX-1 for baseline prostaglandin synthesis, which is constitutive, and COX-2, which is induced under specific conditions [23]. Tissue-specific prostaglandin synthases determine the creation of specific end products, like prostaglandins (prostaglandin D2 (PGD_2_), prostaglandin E2 (PGE_2_)) and prostacyclins (PGI_2_). Thromboxanes are produced by these products, which originate from the COX pathway (through COX-1 and COX-2) while lipoxygenase (LOX) pathway generates leukotriene (LTB_4_), cysteinyl-leukotrienes (leukotriene C4 (LTC_4_), leukotriene D4 (LTD_4_), leukotriene E4 (LTE_4_), hydroxycosatetraenoic acids (HETE), like 5-hydroxycosatetraenoic acid (5-HETE), 12-hydroxycosatetraenoic acid (12-HETE), and 15-hydroxycosatetraenoic acid (15-HETE), and epoxycosatrienoic acids (EET) [20].

Eicosanoids’ physiological effects are determined by how their receptors are distributed and expressed in cells and tissues, which then activate signalling pathways [20,23].

Several cleavage variants have been found in nine G protein-coupled receptors (GPCRs). All of these receptors, which are known as P-receptors, are named for the first letter of their natural ligand. PGD_2_ utilizes D-type prostanoid receptor 1 (DP1) and D-type prostanoid receptor 2 (DP2), and PGE_2_ uses prostaglandin E2 receptor 1 (EP1), prostaglandin E2 receptor 2 (EP2), prostaglandin E2 receptor 3 (EP3), and prostaglandin E2 receptor 4 (EP4). Specific receptors correspond to prostaglandin F_2_α (prostaglandin F receptor (FP)), prostacyclin or PGI_2_ (prostacyclin receptor (IP)), and thromboxane A_2_ (thromboxane prostanoid receptor (TP)) [20]. Traditionally, prostaglandin receptors activate distinct signalling pathways based on their coupling to G-proteins: IP, DP1, EP2, and EP4 typically bind to Gs proteins to increase intracellular cyclic adenosine monophosphate (cAMP); EP1, FP, and TP receptors bind to Gq proteins, resulting in increased intracellular calcium; and EP3 isoforms commonly bind to Gi proteins, resulting in reduced cAMP levels [24].

TXA_2_, a key vasoconstrictor and pro-aggregatory eicosanoid, is primarily made by platelets but also to some extent by ECs. The main natural ligand for TP receptors, TXA_2_, exhibits high affinity; however, at high concentrations, other lipid mediators, including specific prostaglandins, isoprostanes, and HETEs, can also activate TP receptors, thus causing blood vessel muscle contraction [25].

Vascular ECs mainly produce PGI_2_, a key AA by-product, and platelets also contribute; vasodilation by PGI_2_, through IP receptors, is crucial for cardiovascular health. The IP’s binding causes activation of membrane-bound adenylyl cyclase, which then generates the second messenger cyclic AMP (cAMP). This intracellular messenger controls cell responses, such as stopping platelets from clumping, relaxing blood vessels, and increasing thrombomodulin (TM), a protein that prevents coagulation [26].

Conversely, PGF2α is a potent vasoconstrictor, increasing blood pressure and atherosclerosis through activation of the FP receptor [25].

**Figure 1:**
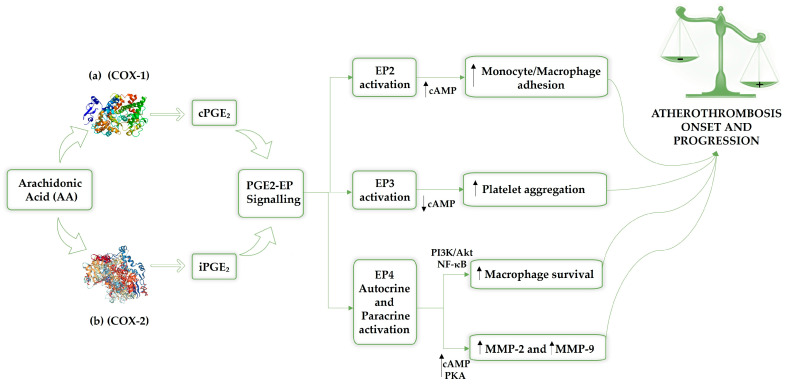
The release of arachidonic acid from membrane phospholipids by cytosolic phospholipase A2 (cPLA2) is promoted by stimulation from factors such as mechanical injury, cytokines, or growth signals. Consequently, arachidonic acid is transferred to prostaglandin H synthase (PGHS), also known as cyclooxygenase, which exhibits two isoforms: COX-1 (**a**) is responsible for basal prostaglandin synthesis (constitutive), and COX-2 (**b**) is induced under specific conditions. Prostaglandin E2 (PGE2) is the prevalent prostaglandin in the human body and contributes to the development of atherosclerosis, exerting its effects through a variety of EP receptors involved in the formation and stabilization of atherosclerotic lesions. Therefore, PGE2-EP signalling could be a promising therapeutic strategy for atherothrombosis. Specifically, the activation of the EP2 receptor promotes monocyte/macrophage adhesion to vascular endothelial cells, a mechanism associated with the inflammatory process involved in the progression of atherothrombosis. The activation of the EP3 receptor, which is followed by the inhibition of the cAMP-dependent pathway, results in the promotion of platelet aggregation and the development of atherothrombosis, which is mediated by PGE2. EP4 modulation involves both autocrine and paracrin activation; indeed, EP4 receptor activation promotes macrophage survival through the PI3K/Akt and NF-κB signalling pathways. Furthermore, EP4 receptor activation stimulates the cAMP-PKA pathway, enhancing the production of MMP-2 and MMP-9 in macrophages of symptomatic atherosclerotic plaques, which leads to plaque instability. AA: arachidonic acid; COX-1: cyclooxygenase 1; COX-2: cyclooxygenase 2; cAMP: cyclic adenosine monophosphate; cPGE2: constitutive prostaglandin E2; iPGE2: inducible prostaglandin E2; PGE_2_: prostaglandin E2; EP2: prostaglandin E2 receptor 2; EP3: prostaglandin E2 receptor 3; EP4: prostaglandin E2 receptor 4; PI3K/Akt: phosphatidylinositol 3-kinase/protein kinase B; MMP-2: metalloproteinases 2; MMP-9: metalloproteinases 9.

The main prostaglandin, PGE_2_, affects atherosclerosis development through multiple E-type prostanoid receptors (EP), which are involved in creating and maintaining atherosclerotic lesions, indicating that PGE_2_-EP signalling may offer a novel treatment for atherosclerosis [25]. Although the EP1 receptor’s function is unclear, stimulating the EP2 receptor supports the adhesion of monocyte/macrophages to vascular ECs, which contribute to atherosclerosis. Activation of EP3 receptors and the cAMP-dependent pathway’s inhibition by PGE_2_ encourages platelet aggregation, leading to atherothrombosis. Conversely, macrophage survival is supported by EP4 receptor activation via the phosphatidylinositol 3-kinase/protein kinase B (PI3K/Akt) and nuclear factor-κB (NF-κB) signalling pathways [20,27,28,29,30,31,32], as shown in Figure 1.

By activating the EP4 receptor, PGE2 may also increase metalloproteinases 2 (MMP-2) and metalloproteinases 9 (MMP-9) production by triggering the cAMP-PKA pathway [32].

In human atherosclerotic lesions, EP4 is the primary PGE_2_ receptor, and too much of it worsens the inflammatory response in plaques [33]. Clinical findings from the last three decades underscore the importance of eicosanoids, including PGI_2_, TXA_2_, and PGE_2_, in vascular system diseases. Analysis involves their interaction with specific receptors and the intracellular pathways regulating inflammation and endothelial function, emphasizing their dual prothrombotic and pro-inflammatory roles compared to protective factors in various cardiovascular diseases [34].

### Objectives

This scoping review aims to provide a comprehensive evaluation of the evidence on the role of eicosanoids and their receptors in platelet and vascular dysfunction involved in atherothrombosis. Specifically, it seeks to accomplish the following:

1. Examine the contribution of eicosanoids and their receptors to platelet and endothelial dysfunction, as well as vascular inflammation, which represent key mechanisms underlying atherothrombotic disease.
2. Assess how different eicosanoids and their receptors regulate prothrombotic and antithrombotic mechanisms.
3. Identify potential therapeutic targets by synthesizing current evidence on eicosanoid receptor pathways that could be modulated to prevent or treat atherothrombosis.

The goal of this review is to synthetize and integrate existing knowledge to support the development of innovative therapeutic strategies targeting eicosanoid receptor signalling in atherothrombotic disorders.

## 2. Methods

We followed the PRISMA-ScR (Preferred Reporting Items for Scoping Reviews and Meta-Analyses extension for Scoping Reviews) guidelines [35].

### 2.1. Eligibility Criteria

#### 2.1.1. Inclusion Criteria

Because of limited findings, we used different study types and broad criteria to create a comprehensive overview. Indeed, eligible studies included human research studies, with no restrictions on age, sex, or clinical context. We also included randomized clinical trials, meta-analyses, and review articles, as these study designs offer higher levels of evidence and allow for the synthesis of clinical findings, potentially clarifying the molecular pathways underlying the interaction between different eicosanoids and related receptors. To gain mechanistic and translational insights, in vivo and in vitro studies were also evaluated. Only full-text articles published in English were considered, and the search was limited to studies published between January 2005 and July 2025, in order to include both basic research and the most recent discoveries in the field.

Although non-human studies were not explicitly listed among the inclusion criteria, the consideration of research based on animal experiments indicates that studies involving non-human subjects were inadvertently included in this review.

This review enabled us to examine every pertinent piece of evidence.

#### 2.1.2. Exclusion Criteria

Studies were not included if they did not meet basic methodological quality criteria or were not directly relevant to the research question. To maintain consistency in data extraction and synthesis, only English-language publications were considered eligible. Articles published outside the specified time period or regarded as outdated were excluded, given that we analysed a field of ongoing research. In particular, studies initially focused on the molecular processes that control the COX-2 inflammatory pathway, whereas studies on eicosanoid receptors’ genetic, epigenetic, and expression modulation have increased in the past two decades. Studies without full-text access were removed, as incomplete reporting prevents a complete evaluation of methodologies and findings. The anecdotal nature and poor generalizability led to the exclusion of case reports. Also, due to their descriptive or narrative style and absence of scoping methods, sources like books and book chapters were not considered. Preprints were omitted to avoid including unverified research.

### 2.2. Search Strategy

This scoping review investigated the impact of eicosanoid–receptor interactions on prothrombotic and antithrombotic mechanisms, specifically concerning endothelial damage, platelet dysfunction, and vascular inflammation. The purpose was to find effective ways to treat and prevent atherothrombosis.

**Figure 2:**
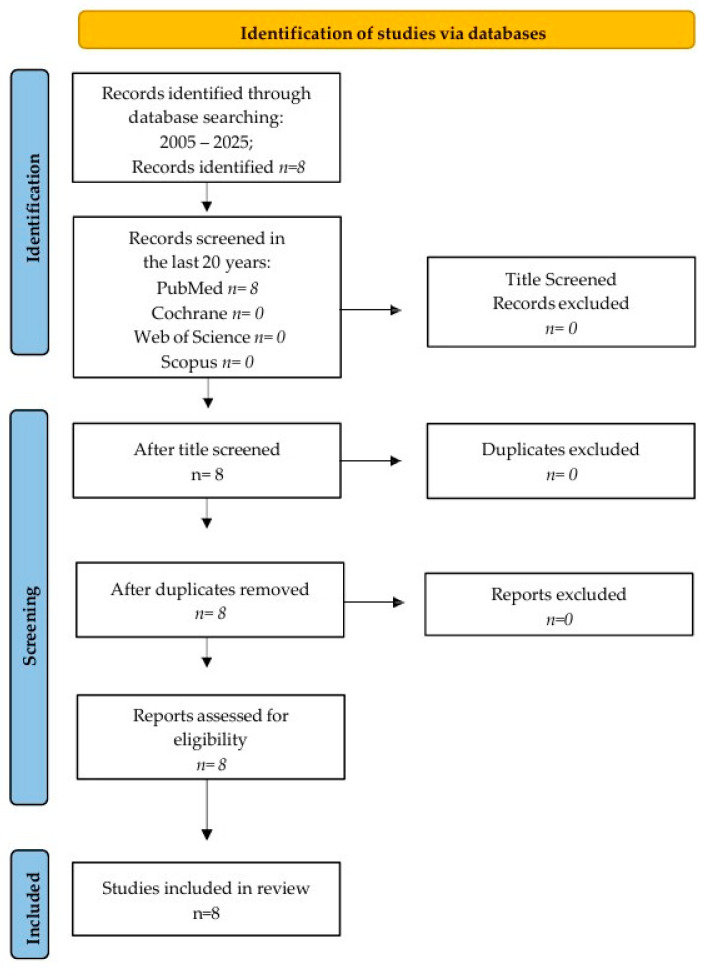
Prisma flow chart.

This scoping review used four different research databases, which included PubMed, Web of Science, Cochrane, and Scopus, to find studies, following the PRISMA-ScR (Preferred Reporting Items for Scoping Reviews and Meta-Analyses extension for Scoping Reviews) guidelines (Figure 2) and the Population Intervention Comparison Outcome Population (PICO) framework. The criteria included all English articles published from January 2005 to July 2025. Articles published in the last 20 years were chosen for this review’s qualitative analysis. The review protocol was registered with OSF (Open Science Framework), available at https://doi.org/10.17605/OSF.IO/WGHFT.

We use the following search terms: “eicosanoids, eicosanoid receptors, platelet dysfunction, vascular dysfunction, and atherothrombosis”.

### 2.3. Screening and Selection of Studies

For processing, all records retrieved from PubMed, Cochrane, Web of Science, and Scopus were first imported into Microsoft Excel. Duplicate entries were detected and eliminated based on their DOIs. Two reviewers independently conducted the screening in two phases.

Initially, we reviewed titles and abstracts to find studies about eicosanoids, receptors, and their impact on atherothrombosis. If they explored molecular mechanisms and receptor expression in atherothrombosis, they were considered for eligibility, regardless of the model or design. Records that met these criteria were moved forward for full-text review.

The second stage involved checking entire articles against set standards, such as being published in English between January 2005 and July 2025, relating to atherothrombosis pathophysiology, and including eicosanoid and receptor biology data. The research incorporated RCTs, meta-analyses, and scoping reviews, with original research articles also included. Studies were removed if their full texts were not available, if they were not peer-reviewed, or if they were outside the time frame. We also excluded case reports, book chapters, preprints, and non-English publications. Reviewer disagreements were settled via conversation and agreement.

### 2.4. Data Charting

Data were extracted separately by two reviewers with a protocol-developed and tested reporting form. Iterative changes to the form ensured the clear and consistent documentation of bibliographic references. The retrieved information consisted of bibliographic details, including authors, year of publication, country, and journal, as well as study characteristics, for example, design, experimental type, and the population or model that was studied. Methodological aspects, such as outcome measures, were also documented. The key results were documented, with a focus on how eicosanoid–receptor interactions affect platelet/endothelial dysfunction.

#### 2.4.1. Collating, Summarizing, and Reporting the Results

Extracted data were gathered and synthesized in accordance with the core research questions.

The first objective involved summarizing the shared mechanisms across several studies. This involved receptor–eicosanoid interactions affecting platelet aggregation, vascular permeability, and inflammatory pathways.

The second aim revealed opposite results: encouraging platelet activation and thrombus formation, but also adjusting protective pathways.

Regarding the third objective, we emphasized receptors and pathways that could be targets for drug modification.

#### 2.4.2. Consultation Exercise

The preliminary results were reviewed with several specialists, including pharmacology and cardiovascular experts, to ensure summary validity. We used these discussions to improve our understanding of receptors and ensure the evidence matched the latest findings. This consultation strengthened the reliability of the graphic data and the presentation of results.

## 3. Results

A literature search conducted from January 2005 to July 2025 found eight articles on PubMed, with no matches found on Scopus, Web of Science, or Cochrane. Screening the titles resulted in eight articles being identified. All eight articles were assessed for eligibility after screening the abstracts, as there were no duplicates. Finally, the eight articles were included in the qualitative analysis.

In summary, the key aspects of the eight studies investigating eicosanoid–receptor interactions and vascular and endothelial dysfunction in the development of atherothrombosis are presented in Table 1.

The studies included in this scoping review emphasize the pivotal role of eicosanoids and their receptors in the pathogenesis of atherothrombosis through a multitude of molecular mechanisms. Various signalling pathways, including specific eicosanoids, enzymes, and other regulatory molecules, regulate the equilibrium between prothrombotic and antithrombotic states. A better understanding of these molecular targets may lead to new therapies, which could prevent atherothrombosis and lessen the side effects of current treatments.

Recently, the key role of eicosanoid pathways in both the growth and evolution of atherothrombotic disease was highlighted. The complex and frequently contradictory control mechanisms of eicosanoid signalling have, in part, limited therapeutic targeting of eicosanoid receptors. Despite advancements in research using knockout mouse models, human genetic analyses, including genome-wide association studies (GWASs), and identification of key genes and proteins, traditional cardiovascular risk factors remain the most significant predictors of disease risk. Nonetheless, natural mutations provide opportunities to replicate knockout genes in humans, providing important insights into gene functions and disease processes. New findings suggest that single mutations frequently cause structural changes in proteins, resulting in unique and often unpredictable downstream signalling changes.

In this context, studying these dysfunctional receptor variants could help us better understand receptor biology. An important future goal is to determine if directly targeting downstream eicosanoid receptors offers extra therapeutic advantages in a cost-effective way for treating and preventing atherothrombotic disease. Accomplishing this requires a thorough analysis of receptor structure and function within specific tissues, potentially involving the creation of ligands that selectively target conformations to finely control eicosanoid signalling at GPCRs [36]. A study revealed that a natural prostacyclin receptor mutation (R212C) failed to activate adenylyl cyclase in clinical and in vitro CV-1 in Origin, Simian, SV40-transformed (COS-1) upregulation system, disrupting signalling, which increases platelet aggregation and promotes atherothrombosis. In three Caucasian cohorts, R212C did not cause cardiovascular disease, but it did negatively impact high-risk subjects, increasing cardiovascular events through the promotion of key atherothrombosis factors. Its adverse impact depended on the presence of vascular injury or atherosclerosis. On the whole, insufficient human prostacyclin receptor (hIP) signalling is a factor in the negative effects of COX-2 inhibitors on the heart. Despite the low frequency of the R212C hIP polymorphism, 2% of the over 60 million cardiovascular patients in the US translates to over 1 million patients [52].

A further study examined the dimerization of the prostacyclin receptor (IP), IPR212C, and the thromboxane A2 receptor alpha isoform (TPα) in HEK293 cells. IPR212C produced standard homo- and hetero-dimers; however, cAMP generation was reduced and endoplasmic reticulum (ER) localization increased. If co-expressed alongside wild-type IP, IPR212C displayed a dominant negative effect, inhibiting wild-type IP signalling and ER trafficking; TPα signalling was also normalized by dimerization with IPR212C. The negative effect probably explains how a variant prostacyclin receptor could amplify platelet loss and enhance cardiovascular disease [53].

Recent evidence showed that the IP and TPα receptors, PGI_2_ and TxA_2_, interact functionally due to constitutive heterodimerization. Although each receptor has standard signalling (IP to cAMP, TP to inositol phosphates), TP activation creates cAMP only with IP co-expression, and this effect is missing in IP-deficient cells. The IP agonist cicaprost increases this TP-dependent cAMP response without involving direct IP signalling; thus, IP presence is needed, though not its activation. Additionally, iPE2III, an isoprostane that does not bind well to isolated receptors, triggers significant cAMP production in IP/TPα-expressing cells, implying the creation of a novel ligand-binding site within the heterodimer. IP/TPα dimerization redirects TP signalling to a PGI_2_-like cAMP response, showing a novel method by which IP can limit TP-mediated cell actions [54].

A low occurrence of hIP variants often restricts pharmacogenetic analyses. A study of 1761 people revealed 18 non-synonymous variants, with eight disrupting receptor binding, activation, or protein stability; notably, M113T, L104R, and R279C exhibited significant misfolding. Non-synonymous mutation carriers showed higher rates of major coronary artery obstruction compared to those without cAMP reduction and controls in case–control analyses. These results highlight the heart-protective effect of regular hIP signalling via cAMP and demonstrate the value of combining in vitro functional assays with human genetic studies for predicting clinical results. Consequently, normal signalling (via cAMP) at nm concentrations of hIP agonist is key for cardioprotection. The limitation of the study is its small size, especially for low-prevalence mutations. Missing mutations are an additional issue, along with in vitro and in vivo testing. Therefore, confirmation on a larger scale is required [55].

Table 2 summarizes the main research studies that highlighted dysfunctional eicosanoid receptor variants involved in atherothrombotic risk factors.

According to research, prostaglandin E2, important for healthy blood vessels, enhances platelet clumping, which is modulated by EP3, when stimulated moderately.

Specifically, an in vitro study found that the EP3 receptor’s activation on platelets by low PGE_2_ concentrations enhanced their agonist sensitivity and enabled full aggregation, even with submaximal stimulation. The results imply that enough PGE_2_ is released to increase platelet activation from adenosine diphosphate (ADP), TXA_2_, and/or collagen following arterial wall damage. Observation of PGE_2_ synthesis in atherosclerotic plaques led to the conclusion that the EP3 receptor is not activated when PGE_2_ production is inhibited, which means the pro-aggregative effect of plaque homogenates is caused by PGE_2_.

PGE_2_ from atherosclerotic plaques activates the EP3 receptor, according to these findings. Preventing this pathway requires stopping PGE_2_, which has diverse functions in the body, so suppressing its creation could lead to unforeseen side effects [27].

EP3 receptor inhibition, on the other hand, may be safer, especially considering that the EP3 receptor has three isoforms.

More specifically, the EP receptor has two isoforms, namely, prostaglandin E2 receptor 3 alpha (EP3α) and prostaglandin E2 receptor 3 beta (EP3β), which differ in their carboxy-terminal sequences. These are a result of alternative splicing and vary in how well they activate the inhibitory G protein and in their ability to inhibit adenylate cyclase. Through cloning of the two EP isoforms, a third isoform emerged, which couples both the stimulation and inhibition of adenylate cyclase, highlighting the dual molecular effect on the messenger [56].

Therefore, a treatment specifically targeting the platelet EP3 receptor, which controls the suppression of adenylate cyclase, could be more targeted than medicines that broadly inhibit PGE_2_ production. For example, low-dose aspirin, which is standard treatment, inhibits the activity of COX-1 but not COX-2, which is responsible for PGE_2_ production in atherosclerotic plaques [27].

Platelet clumping and blood clots are mainly caused by TXA_2_ and its TP receptors. Indeed, different evidence highlighted that TP receptors contribute to vascular wall dysfunction, including impaired endothelium-dependent vasodilation.

These unhealthy processes are lessened by TP receptor antagonists, which protect the vascular wall. Furthermore, ROS production and accumulation stabilize and increase TP receptor expression and also reduce NO production in ECs. These effects together cause vasoconstriction and thrombosis [41]. Furthermore, the activation of TP receptors could be directly involved in the chronic inflammation that contributes to the advancement of atherosclerotic vascular disease. The significance of TP receptor stimulation’s effect on vascular cell adhesion molecule 1 (VCAM-1) expression was shown in a study that examined how the activation of TPr by U46619 amplifies interleukin-1 beta (IL-1β)-induced VCAM-1 expression in vascular smooth muscle cells (VSMCs). U46619 alone was found to have no impact on NF-κB activation or on the induction of VCAM-1 expression. However, when NF-κB was activated by interleukin-1 beta (IL-1β) or U46619, U46619 amplified VCAM-1 expression through the c-jun N-terminal kinase (JNK) pathway, which then stimulated Activator Protein 1 (AP-1) and VCAM-1 gene transcription in both rat and human VSMCs, possibly explaining why TPr activation contributes to vascular disease development [40].

A study on mice lacking apolipoprotein E (Apo-E) and TP receptors suggested that atherosclerosis development benefits from TP receptor blockade. Due to the effect of TP receptors on endothelial function and inflammation, blocking TP receptors may be useful in fighting accelerated atherosclerosis in hyperglycaemia and diabetes models. Indeed, mice lacking Apo-E (apoE−/−) with induced type 1 diabetes had at least a 3-fold increase in atherosclerotic lesions [38,39].

Specifically, a study suggested that blocking the TP receptor could help treat accelerated atherosclerosis in diabetic patients, showing a greater impact in diabetic apoE−/− mice, which nearly reversed the processes speeding up atherosclerosis. Indeed, S18886, a potent TP inhibitor, effectively blocked the diabetes-related rise in lesion area, while not impacting hyperglycaemia or hypercholesterolaemia, and further prevented the decline in endothelial function and endothelial nitric oxide synthase expression, alongside the diabetes-linked increase in inflammation markers.

The positive outcome may be linked to the prevention of TP-related damage to eNOS expression, endothelial function, and the increase in inflammatory gene expression and oxidative stress when endothelial cells respond to diabetes [38].

These cellular and animal studies indicate that TP receptor antagonists could treat vascular disease through effects beyond just preventing blood clots, including actions on vascular adhesion molecules, eNOS regulation, and lessening oxidative stress.

Consequently, TP receptor antagonists are a potentially fruitful approach for preventing vascular disease, partly because of these pleotropic effects beyond their antithrombotic activities [37].

Atherosclerosis causes higher levels of TXA_2_ and prostacyclin. To investigate this, a study bred apoE-deficient mice (apoE−/−), prone to atherosclerosis, with mice lacking TP or IP. ApoE–/–TP–/– mice showed delayed atherogenesis, while apoE–/–IP–/– mice showed accelerated atherogenesis compared to apoE-deficient mice (apoE−/−). Compared to apoE–/–TP–/– mice, plaques in apoE–/–IP–/ mice presented partial endothelial rupture, with elevated Intercellular Adhesion Molecule 1 (ICAM-1) and reduced Platelet Endothelial Cell Adhesion Molecule-1 (PECAM-1) expression. Compared to apoE–/– mice, ex vivo thrombin-induced platelet activation revealed greater P-selectin expression on platelets from apoE–/–IP–/– mice and lower P-selectin expression on platelets from apoE–/–TP–/– mice. The results proved that TXA_2_ supports and PGI2 hinders the start and progression of atherogenesis by regulating platelet activation and the interaction between leukocytes and endothelial cells [39].

Table 3 summarizes the main research studies that highlighted TP receptor modulation and the related endothelial inflammation.

By encouraging re-reendothelialization and limiting vascular remodelling, endothelial progenitor cells (EPCs) aid vascular repair because neointimal hyperplasia is a common feature of angioplasty and atherosclerosis. EPCs without the IP receptor showed reduced adhesion, migration, and proliferation, but IP agonists enhanced these functions only in wild-type cells. In a mouse injury model, removing IP from bone marrow cells caused increased neointimal hyperplasia. Furthermore, wild-type (WT) EPCs, not IP-deficient EPCs, were recruited effectively to injured vessels and restored typical re-reendothelialization. EPC activity and the control of vascular remodelling depend on the prostaglandin I_2_–IP pathway, as demonstrated by these findings. Even though PGI2’s function in EPC proangiogenesis is documented, its role in reendothelialization is still uncertain [27].

The findings highlighted a new role for PGI_2_ in preventing vascular remodelling; the PGI_2_-IP system controls EPC function, thereby reducing vascular remodelling [44].

PGE_2_ has been shown to exert a biphasic effect on platelet aggregation, enhancing aggregation at low concentrations while inhibiting it at higher concentrations [29].

A study examined the impact of PGE_2_ and its analogue CL 115,347 on human platelet function and blood vessel contraction. PGE_2_ exhibited a two-stage action on platelet clumping, amplifying ADP- and collagen-driven clumping at low levels and suppressing it at high levels, while CL 115,347 had a small effect at low levels and only increased clumping at high levels. Adrenaline-triggered aggregation was stopped by both substances; however, PGE_2_ could boost aggregation with PGI_2_. Platelet-rich plasma and whole blood yielded matching results.

Compared to PGE_2_, CL 115,347 is less strong at controlling platelet activity and blood vessel contraction [29].

By using EP3−/− mice and AE-248, a selective EP3 agonist, a study aimed to define the role of PGE_2_ in platelet function, specifically its impact on haemostasis and thromboembolism. TXA_2_ agonist-induced platelet aggregation was increased by PGE_2_ and AE-248. These compounds also modified intracellular calcium and cAMP; however, this did not occur in EP3−/− mice. EP3−/− mice had extended bleeding, poor clot formation, and better survival after being given arachidonic acid. These findings suggest that PGE_2_, using EP3, is vital for healthy blood clotting and dangerous blood clots. Though species differences can restrict direct human translational application, EP3 shows promise for platelet function therapy in various diseases [28].

Table 4 summarizes the main research studies that highlighted the role of prostanoids in vascular remodelling and platelet dysfunction.

Compared to wild-type mice, EP3-deficient (EP3−/−) mice had longer bleeding times and a lower death rate after acute thromboembolism caused by AA. Additionally, in a model of mechanical plaque disruption, PGE_2_ generated within atherosclerotic lesions was shown to promote atherothrombosis via EP3 signalling in platelets. This effect was notably reduced in mice lacking both Apo-E (apoE−/−) and EP3 (EP3−/−). Thus, these results underscore the key function of the PGE_2_–EP3 pathway in thromboembolic events by promoting platelet activation [27,42].

Human atherosclerotic plaques have high levels of MMP-2 (gelatinase A) and MMP-9 (gelatinase B). Collagen digestion and cap weakness rely on these enzymes: MMP-2 and MMP-9 are released by macrophages through a pathway relying on prostaglandin (PG) E2 [32].

Another study explored MMP levels/expression in carotid atherosclerotic plaques, correlating them with clinical symptoms and plaque instability. A total of 75 carotid endarterectomy patients’ plaques were analysed based on their symptom timing. Plaques from symptomatic patients, those embolizing spontaneously, and those histologically unstable showed significantly elevated MMP-9, but not MMP-1, -2, or -3. Increased MMP-9 expression was confirmed by in situ hybridization in inflamed areas. According to the study, high levels of MMP-9 are connected to unstable carotid plaques, implying MMP-9 could be targeted in therapies to stabilize plaques and prevent strokes. A key limitation of the study was the microscopic examination of a small plaque portion, possibly missing key features in some patients [31].

VSMCs inherently produce pro-MMP-2, which is inactive; however, an inflammatory response triggers changes to help secrete every component needed for zymographically active MMP-2 and MMP-9, both enzymes essential for ECM degradation, thus facilitating VSMC growth and migration [57].

So, a large group of Matrix Metalloproteinases (MMPs) operating together could theoretically fully break down the arterial ECM, leading to a thinner fibrous cap and less collagen, typical of plaques that are prone to rupture. Specifically, metalloproteinases 1, 2, 8, 13, and 14 break down strong type-I and type-III collagens, and MMP-9 and -12 break down elastin [58].

As a result, the strong evidence points towards COX-2 expression in macrophages and SMCs, especially when working with mPGES-1, which is crucial for human atherosclerotic plaque to become unstable. Compared to control monocytes, IL-1β-stimulated monocytes showed a significant increase in COX-2/mPGES-1. Over-expression of COX-2/mPGES-1, in particular, led to increased expression of MMP-2 and MMP-9 [46].

Identifying the mechanisms of COX-2 expression in plaque macrophages could assist in developing a key therapy to stabilize human plaques. Based on scientific evidence, a recent study looked at which EP receptor modulates macrophage MMP production in atherosclerotic plaques. The study showed the EP4 receptor alone regulates MMP biosynthesis in vulnerable human plaques via PGE_2_.

Furthermore, carotid lesion plaques that caused recent strokes showed higher EP4 expression compared to samples from asymptomatic patients. It is noteworthy that macrophages with EP4 receptor-positive plaques were also strongly COX-2/mPGES-1-positive, which may have functional significance. The study’s limitation is that it may not apply directly to in-life atherothrombosis, as it lacked in vivo drug tests, and platelets could be key. Aspirin use could have influenced arterial inflammation; however, it probably did not change EP receptor expression differences because use was comparable across groups [46].

The role of COX-2 and PGES in carotid plaques and their connections to inflammation, MMPs, and patient symptoms were the focus of a new study. Symptomatic plaques displayed increased macrophage infiltration and elevated COX-2, PGES, and MMP-2/-9, most notably in plaque “shoulder” areas where they overlapped with activated macrophages. Active MMP forms were also found in these plaques. MMP production was lowered by blocking COX-2 in vitro, but this was restored when PGE2 was added. These findings suggest that plaque instability and acute ischemic events are caused by MMP-mediated plaque rupture, which is caused by the COX-2/PGES activity in activated macrophages [59].

In mice, blocking EP4 in macrophages through genetics and drugs caused cell death and curbed early atherosclerosis by reducing the PI3K/Akt signalling and NF-κB pathways, according to a recent study. This finding reinforces the pro-inflammatory function of EP4 in atherosclerosis [30].

Another study proposed cAMP–protein kinase A (PKA) pathway involvement in EP4 receptor-dependent MMP production. Macrophage incubation with an activator of adenylyl cyclase (forskolin) or a stimulator of PKA (dibutryl cAMP) increased MMP-9 expression [32].

Data suggest that the reduction in COX-2 in inflammatory cells can help prevent plaque instability, but PGI_2_ is key to blood vessel homeostasis [40]. PGI_2_ is a key eicosanoid that relaxes vascular muscles via adenylyl cyclase activation and increased cAMP. PGI_2_ significantly impacts baseline blood vessel tone, crucial for vascular homeostasis. Combined, these functions stress PGI_2_’s key role in blood vessel health, incorporating vasodilation and antithrombosis into the endothelial control system [2].

Table 5 summarizes the main research studies that highlighted the genetic and molecular mechanisms underlying atherosclerotic plaque instability.

In addition, when evaluating human study results, the complexity of COX-2 expression regulation must be highlighted. A combined approach using genetic, biochemical, and pharmacological methods could provide further mechanistic insights. This may define the causes of cardiovascular response to COX-2 inhibitors and discover fresh targets for drug treatment that precede or follow COX-2 expression.

The original purpose of COX-2 inhibitors was to address the gastrointestinal problems of non-steroidal anti-inflammatory drugs (NSAIDs); however, they can also contribute to atherothrombosis by inhibiting PGI_2_, which prevents platelet aggregation. The role of this mechanism is especially critical in diseases with increased COX-2 expression, like atherosclerosis. Furthermore, selective COX-2 inhibitors do not prevent platelets from synthesizing TXA_2_; therefore, the increased risk of thrombosis may be due to the unopposed effects of TXA_2_ on platelets. However, prostaglandins could also fight thrombosis through means other than stopping platelet function, such as changing gene expression in vascular cells. Using microarray technology, iloprost (a stable PGI_2_ analogue) treatment has been shown to elevate TM mRNA expression in cultured human coronary artery SMCs [49].

TM mainly resides on ECs and acts as a thrombin receptor. Activated protein C (aPC) is key to controlling blood clotting by stopping thrombin generation via factors Va and VIIIa. A study’s findings indicated an enhancement in the expression of functionally active TM protein in cultured human SMCs by PGI_2_ mimetics. Notably, research has demonstrated that prostaglandins produced via the COX-2 pathway control both the gene expression and function of TM. Indeed, immunohistochemical analysis of atherectomy samples extracted from human carotid arteries revealed that COX-2 and TM are co-expressed in SMCs within atherosclerotic lesions. TM expression is regulated by COX-2 prostaglandins. Given that thrombomodulin inhibits blood coagulation, this interaction may offer a novel, platelet-independent mechanism that could explain, at least partially, the prothrombotic phenotype seen after COX-2 inhibition. These findings point to a new pathway, independent of platelets, where TM prevents blood clotting, which may clarify the prothrombotic actions of COX-2 inhibitors [48]. Additionally, targeting the impact of prostaglandins from the COX-2 pathway on TM gene expression and its anticoagulant function may be a future therapy.

The PTGIR gene encodes the hIP protein, which is a G protein-coupled receptor (GPCR) from the class A rhodopsin-like family characterized by seven transmembrane domains. PTGIR gene polymorphisms are a useful model for studying how PGI_2_ signalling may cause atherosclerosis and thrombotic disorders in humans.

A study was conducted to investigate whether polymorphisms in the PTGIR gene could increase susceptibility to DVT. In a group of DVT patients and healthy individuals, the connection between PTGIR SNPs and DVT was investigated, along with systemic markers of vascular issues and platelet activation, namely, sP-selectin and urinary 11-dehydro-TXB_2_. Significantly, sP-selectin and 11-dehydro-TXB_2_ levels were increased in patients with DVT compared to controls, but only in individuals exhibiting the synonymous PTGIR variants V53V and S328S. This implies a possible relationship between these polymorphisms and elevated platelet activation in DVT. Furthermore, R212C, a non-synonymous variant, was found to be a dysfunctional PTGIR polymorphism linked to intimal hyperplasia. Despite the small sample size, the study’s results strongly suggest the importance of human IP in vein and artery diseases, showing varied effects from genetic differences. Therefore, a comparative data analysis, supported by the literature review, underscores the impact of PTGIR polymorphisms on thrombosis and atherogenesis development [26].

A study was conducted to investigate how COX-1, COX-2 inhibitors, and naproxen affect coronary blood vessel function and clotting under normal and LPS-induced inflammation.

The findings emphasized how endothelial COX-2 induction is key to maintaining vascular equilibrium by shifting the balance of prostanoids from prothrombotic to antithrombotic.

According to the in vivo study, COX-2 activation in the endothelium boosts PGI_2_, not TXA_2_, which indicates an anti-clotting effect. Despite their safety for healthy people, COX-2 inhibitors can raise the chance of blood clots, especially in those with inflammatory diseases like vasculitis [51]. Unlike conventional NSAIDs, COX-2 inhibitors do not stop platelet COX-1 from making TXA_2_, which can interfere with TXA_2_/PGI_2_ balance, possibly causing blood clots. COX-2 is not found in typical blood vessels, but it is overproduced in atherosclerotic plaques; its activation is triggered by issues like inflammation or infection. Moreover, clinical trials have emphasized the cardiovascular dangers of COX-2 inhibition, demonstrating a substantial increase in heart attacks for patients on selective COX-2 inhibitors.

The study’s findings suggest that AA-triggered blood vessel expansion is connected to COX-1’s production of PGI_2_, an effect that can be stopped with certain medications. Furthermore, thrombosis research suggests that in animals given LPS, reactions to vascular injury that promote clotting are self-limiting because of COX-2 and increased PGI_2_. The data imply that the alteration of COX activi.ty was associated with elevated PGI_2_ levels, while TXs remained constant, thus aiding to prevent blood coagulation. However, this protective response can be altered by the administration of a selective COX-2 inhibitor. These outcomes are very significant in light of previously reported cardiovascular complications associated with selective COX-2 inhibitors in clinical trials emphasizing the critical role of inducible vascular COX-2 in preserving vascular homeostasis [50].

## 4. Discussion

This scoping review (January 2005 to July 2025) demonstrates that eicosanoids, their receptors, and vascular/platelet problems are linked to atherothrombosis. Thus, this investigation intends to deliver a thorough analysis of the molecular pathways related to this complex regulatory process [17].

### 4.1. Atherothrombosis and Clotting

Atherothrombosis, in particular, is a complicated and multifaceted disease defined by the chronic inflammatory response that occurs at the vascular wall interface of large and medium arteries. Atherosclerotic plaque develops through lipid accumulation in the tunica intima, its oxidation, attraction of immune cells (mainly monocytes), and subsequent inflammation [36]. The gradual progression of plaque can destabilize it, making it prone to instability or rupture. The interaction between blood and thrombogenic material causes platelet activation and coagulation, ultimately forming occlusive thrombi. Acute ischaemic incidents can arise from the blocking of thrombi, which interferes with blood circulation; therefore, the primary mechanisms causing sudden cardiovascular events are the development and progression of platelet-rich thrombi [60].

### 4.2. Eicosanoids and Atherothrombosis Onset and Development

The role of eicosanoids in human atherothrombosis has only recently been understood. The contrast was obvious when considering the heart-protective effects of low-dose aspirin (COX-1 inhibitor) and the cardiovascular dangers of rofecoxib (COX-2 inhibitor). These findings were further supported by numerous well-designed, informative studies conducted in animal models [61].

The influence of eicosanoids varies depending on their receptors’ locations and expressions, activating internal signals tied to atherothrombosis. Therefore, a primary treatment goal may be to directly stop the downstream signal at the receptor using a blockade or functional modulation. This highlights the need to look at the possible benefit of directly targeting receptors in the advancement of atherothrombosis. We may need to develop conformationally selective ligands to accurately control eicosanoid signalling GPCRs [36].

Genetic variations can further modulate the effects of receptors in this situation, as shown by a study on the hIP protein’s PTGIR gene polymorphisms. Specifically, individuals with the V53V and S328S PTGIR variants showed higher systemic biomarkers of vascular dysfunction and platelet activation, implying a link between these variations and a greater chance of thrombosis and atherogenesis. These findings emphasize how regulating receptor activity is key to managing vascular responses and platelet activation in heart disease [26].

These findings emphasize that receptor activity regulation involves both genetics and extracellular signals. Some prostaglandins, produced by the COX-2 pathway, appear to reduce blood clots through effects on the genes of vascular cells, not by affecting platelets. A particular study examined iloprost, a stable PGI_2_ analogue, which increased TM mRNA expression in human coronary SMCs, implying a protective effect via transcriptional regulation. Notably, data have shown that prostaglandins, produced through the COX-2 pathway, can affect gene expression and TM function via a new platelet-independent pathway, which prevents blood coagulation [48].

### 4.3. The Crucial Role of Prostaglandin E_2_ Receptors in the Two-Stage Processes of Atherothrombosis

Prostaglandin E_2_ receptors (EP1–EP4) are key to transmitting signals for platelet aggregation, inflammation, and immunity. These include the EP3 and EP4 receptors, which are important because they can cause opposing cell responses by influencing distinct signalling pathways. According to new studies, EP3 and EP4 can greatly affect the equilibrium between healthy and unhealthy processes.

It was demonstrated that activating the EP3 receptor on platelets, especially by low PGE_2_ concentrations, results in the inhibition of adenylate cyclase; consequently, this action decreases intracellular cAMP and promotes platelet aggregation.

To verify that EP3 carries out the pro-aggregatory effects of PGE_2_ at low doses, research used EP3-deficient ((EP3−/−)) mice and found that the potentiating effect of low PGE_2_ concentrations on platelet aggregation was completely negated, as EP3 mRNA is the most expressed EP receptor subtype in platelets. In contrast, ONO-AE-248, a selective EP3 receptor agonist, increased platelet aggregation [42].

Targeting the platelet EP3 receptor selectively may be a better therapeutic strategy than non-selective PGE_2_ synthesis inhibition, as PGE_2_ has protective roles in many systems and can counteract the pro-aggregating effect specifically from the PGE_2_-EP3 interaction [36].

EP4 receptor action also significantly affects MMP-2 and MMP-9 creation due to PGE_2_, thereby promoting unstable atherosclerotic plaques. Studies of plaques in human carotid lesions found more EP4 receptor expression. Furthermore, COX-2 expression is amplified by PGE_2_ through a mechanism that EP4 mediates. The evidence points towards a protective effect of lowering COX-2 expression in inflamed cells, potentially preventing the instability and rupture of atherosclerotic plaques. Modifying PGE2–EP4 receptor signalling, like with EP4 receptor antagonists, could be a safer therapy than COX-2 inhibitors for stabilizing plaques in atherosclerosis patients and preventing acute ischemic syndromes.

Targeting EP3 and EP4 receptors, as well as regulating COX-2, could reduce atherothrombotic risk, which may lead to new atherothrombosis treatments. In this regard, recent findings showed that Short-Chain Fatty Acid (SCFA) butyrate intake lessened the expression of the prostanoid EP4 receptor and its involvement in COX-2 production within human colon cancer cells [62]. Gut microbiota-produced butyrate inhibits platelet function by preventing aggregation and offering vascular protection [63,64,65].

Specifically, diet-based butyric acid has a negative relationship with human platelet aggregation, and studies have shown that it inhibits aggregation induced by collagen and other activators. Sodium butyrate also prevents platelet-derived growth factors from multiplying vascular smooth muscle cells, possibly helping to reduce the advancement of atherosclerosis. Despite limited research on the molecular pathways in these anti-atherotrombotic processes, butyrate seems to prevent EP4 overactivation through homeostasis, Interleukin-4 (IL-4), inflammation, and cancer connections [66,67]. By interacting with EP4 receptors on intestinal cells, butyrate maintains balance in healthy guts. A lack of butyrate causes EP4 to become overactive, which can cause excessive inflammation and abnormal cell growth, potentially leading to Inflammatory Bowel Disease (IBD) or colorectal cancer [66]. Furthermore, butyrate promotes Treg cells, which create IL-4, an anti-inflammatory cytokine, thereby diminishing inflammation [67]. These data emphasize that SCFAs may help regulate both inflammation and related thrombotic processes.

Moreover, in addition to butyrate’s main role in the EP4 receptor pathway, oral administration of butyrate to mice with anaphylaxis greatly lessened blood vessel leakage and anaphylactic signs. Administering an EP3 antagonist or a COX inhibitor (e.g., aspirin) eliminated these effects, thus confirming the EP3 receptor’s central role [68]. Furthermore, research suggests that butyrate notably elevates PGE2 production in co-cultures of macrophages and adipocytes. Using an EP3-selective antagonist, it was demonstrated that roughly 40% of butyrate’s inhibition of free fatty acid release is attributed to the PGE2–EP3 pathway [69].

In addition to its role in rapid signalling via the EP3 receptor, butyrate also functions as a Histone Deacetylase (HDAC) inhibitor. This epigenetic process suppresses factors such as the high-affinity IgE receptor (FcεRI) and the Stem Cell Factor (SCF) proto-oncogene, receptor tyrosine kinase (KIT), thereby decreasing mast cells’ sensitivity to allergens over a longer time [70]. These new findings may support the use of EP3 and EP4 modulation in managing the onset and development of various diseases. Nevertheless, more research is required to fully assess how SCFAs impact the molecular processes of endothelial dysfunction. In addition, PGI_2_ has a key physiological function in vascular health [45].

### 4.4. COX-2 and Endothelial Homeostasis

The vascular inducible isoform of COX-2 is crucial for maintaining endothelial homeostasis, which selective COX-2 inhibition does not support. Although COX-2 is poorly expressed in healthy vascular tissue, it is strongly upregulated in atherosclerotic plaques. According to in vivo studies, activating it in the endothelium boosts PGI_2_ production, while TxA_2_ levels remain steady, leading to an antithrombotic effect. Based on these data, preventing thrombogenesis is associated with COX modulation and increased PGI_2_ synthesis versus TxA_2_ [50].

PGI_2_ is both a significant antithrombotic mediator and an essential regulator of vascular remodelling. By stimulating the IP receptor, PGI_2_ reduces smooth muscle cell growth and platelet function while also regulating EPCs, thereby mitigating neointimal hyperplasia following vascular injury [42].

To summarize, many findings show that TP receptors, stimulated by TXA_2_, worsen blood vessel function by hindering vasodilation and causing long-term inflammation via VCAM-1 in muscle cells. Animal model studies have revealed that the TP antagonist S18886 prevents these effects. Beyond blocking platelet activity, TP antagonists could help vascular diseases by managing inflammation, oxidative stress, and endothelial function, which is a hopeful way to keep blood vessels healthy [37].

### 4.5. Limitations

This scoping review has some limitations inherent to the methodology used. In line with PRISMA-ScR standards [35], a formal risk of bias assessment was not conducted, which could impact the depth of interpretation of the results. Based on this, we qualitatively assessed potential risks of bias to help better understand the studies. The database search found a relatively small number of studies, which limits this scoping review. Although all were considered, this highlights the field’s weak evidence. Moreover, several studies have translational limitations, as model results may not represent human pathology. Furthermore, the direct application of these findings to atherothrombosis in human organisms is limited by the lack of pharmacological testing in living systems. This finding may suggest a model validity bias, which is a key point, given that animal models may not mirror human diseases. In addition, this scoping review’s limitations include a small sample size, especially for uncommon mutations, and missing mutations, making data interpretation difficult and suggesting that larger studies are required. Furthermore, microscopic analysis of atherosclerotic plaques constitutes another significant limitation, potentially overlooking critical pathological features in some patients. The results of this scoping review should be interpreted keeping in mind that a rigorous risk of bias assessment, in line with the methodological standards of scoping reviews, was not conducted. Therefore, these conclusions do not assess the quality of the evidence, but merely describe its extent and nature.

## 5. Conclusions

In conclusion, this scoping review analysed the evidence on the role of eicosanoids and their receptor subtypes in pro- and antithrombotic pathways. The relationship between these pathological processes is essential to the mechanisms of atherothrombosis, which involves dysfunctional platelets, endothelium, vessels, and inflammation. The ultimate goal of this review was to identify potential therapeutic targets for preventing and treating atherothrombotic disease. The current evidence suggests a promising therapeutic strategy for cardiovascular dysfunctions: targeted modulation of eicosanoids and their receptors. Modulating these pathways could have an impact on a range of critical pathophysiological processes, such as platelet activation, coagulation, inflammation, oxidative stress, and endothelial function. To lower thrombotic risk and enhance atherosclerotic plaque stability, which offers new approaches to prevent and treat atherothrombosis, it may be appropriate to consider targeting receptors like EP3, EP4, and TP, alongside regulating COX-2 activity.

Based on these findings and the lack of comprehensive results from the scientific database searches performed, it appears appropriate to promote additional in vitro, in vivo, and clinical studies.

Further experimental investigations could provide more detailed explanations and concrete evidence, establishing comprehensive knowledge for future research and developments.

### OSF Registration Statement and Number

The protocol has been registered with the OSF (Open Science Framework), available from https://doi.org/10.17605/OSF.IO/WGHFT.

## Figures and Tables

**Table 1:** The selected articles, specifying the aims, databases used, and results.

Authors, Year	Aim of Studies	Search Databases	Types of Studies Included	Summary of Results	Refs.
Gleim S, et al. 2012	To emphasize the connection between eicosanoids and heart health risks, focusing on eicosanoid receptor function and genetic mutations.	PubMed	Review	Examining spontaneous dysfunctional variants of eicosanoid receptors is a good way to improve receptor biology. It may offer more therapeutic advantages. This approach is also cost-effective for treating and preventing atherothrombotic disease.	[36]
Gross S, et al. 2007	To determine whether PGE_2_ affects TXA_2_-induced thrombosis in mice without EP3.	PubMed	Comparative study	PGE_2_, released from atherosclerotic plaques, increases platelet activation in the arteries by activating the EP3 receptor when the arterial wall is damaged.	[27]
Félétou M, et al. 2010	To describe how using thromboxane receptor blockers in animal models protects blood vessels, emphasizing the part that eicosanoid levels play in vascular problems, inflammation, and atherothrombosis.	PubMed	Review	TP receptor inhibition, besides its anti-platelet effect, could be especially effective in treating atherosclerosis by modulating chronic inflammation and oxidative stress, which may have anti-atherogenic benefits, especially in diabetes.	[37,38,39,40,41]
Yuhki K, et al. 2011	To investigate knockout mice and show that prostanoids regulate platelets, which is important in cardiovascular diseases such as atherosclerosis.	PubMed	Review	Platelet aggregation is increased by PGE_2_ at low levels, which functions by activating the EP3 receptor, a process that is not present in EP3-deficient platelets. EP3 pathways contribute to atherothrombosis after plaque rupture, emphasizing its role in heart disease.	[27,28,29,39,42,43,44]
Cipollone F, et al. 2010	To analyse the molecular mechanisms and immune cells in destabilizing atherosclerotic plaques and discover treatments to stabilize vulnerable plaques.	PubMed	Review	In symptomatic atherosclerotic plaques, the EP4 receptor stimulates macrophages to produce MMP-2 and MMP-9 in response to PGE_2_, leading to plaque instability through a mechanism linked to the inflammatory cyclooxygenase 2/microsomal prostaglandin E synthase-1 (COX-2/mPGES-1) pathway. The data imply that when COX-2 expression is reduced in inflammatory cells, the likelihood of plaque rupture and instability decreases.	[45,46,47]
Rabausch K, et al. 2005	To show that prostaglandins, which are produced internally via the COX-2 pathway, can trigger the expression of active TM in human SMCs.	PubMed	Comparative study	Prostaglandins are found to regulate TM gene expression and activity, uncovering a new platelet-independent pathway that inhibits blood clotting, which may explain the prothrombotic effects of COX-2 inhibitors.	[48,49]
Patrignani P, et al. 2008	To assess any differences in systemic markers of vascular disease and platelet activation and the influence of Prostaglandin I2 Receptor (PTGIR) gene polymorphisms.	PubMed	Research article	Patients with deep vein thrombosis (DVT) and platelet activation risks present unique PTGIR V53V/S328S polymorphisms. Furthermore, a dysfunctional PTGIR polymorphism (R212C) is found to be connected to intimal hyperplasia.	[26]
Hong TT, et al. 2008	To evaluate how COX-1 and COX-2 inhibitors, plus naproxen, a non-selective COX inhibitor, affect coronary vasodilation and thrombogenicity during normal and Lipopolysaccharide (LPS)-induced inflammation.	PubMed	Research article	Variations in COX activity, combined with increased PGI_2_ production but not thromboxanes (TXs), create an effect preventing blood clotting, highlighting COX-2’s role in vascular health.	[50,51]

COX-1: cyclooxygenase 1; COX-2: cyclooxygenase 2; DVT: Deep vein thrombosis; EP3: prostaglandin E2 receptor 3; EP4: prostaglandin E2 receptor 4; LPS: Lipopolysaccharide; MMP-2: metalloproteinases-2; MMP-9: metalloproteinases-9; mPGES-1: microsomal prostaglandin E synthase-1; PGE_2_: prostaglandin E2; PTGIR: Prostaglandin I2 Receptor gene polymorphisms; SMCs: smooth muscle cells; TP: thromboxane prostanoid receptor; TXA_2_: thromboxane A_2_; TXs: thromboxanes.

**Table 2:** Dysfunctional eicosanoid receptor variants involved in atherothrombotic risk factors. ↑: Increase; ↓: decrease.

Authors, Year	Review	Types of Studies Included in Review	Aim of Studies	Summary of Results	Ref.
Gleim S, et al. 2012 [36]	(An eicosanoid-centric view of atherothrombotic risk factors)	Human genetic association study and in vitro functional study	In the high-risk cardiovascular group, the prostacyclin receptor variant (R212C) is unable to activate adenylyl cyclase in patient blood and an in vitro COS-1 over-expression system.	***Human studies:*** ↑ Platelet aggregation. ↑ Both disease severity and adverse cardiovascular events in patients with R212C variants, with respect to normal allele patients. ↑ Disease progression in patients with greatest risk factors. **In vitro *studies:*** Defective hIP signalling, caused by R212C, represented a key factor in atherothrombosis onset and progression.	[52]
		In vitro study	To evaluate the incorporation of IP^R212C^ into homo- and hetero-dimeric receptor complexes and its role in the responsiveness of platelet IP and the enhancement in cardiovascular disease progression.	**In vitro *studies:*** ↓ cAMP generation by IPR212C. ↑ ER localization, but underwent normal homo- and heterodimerization. ***Human studies:*** ↓ Platelet IP responsiveness. ↑ Cardiovascular disease in individuals heterozygous for an IP variant, IP^R212C^.	[53]
		In vitro study	To determine if the interaction between PGI2 and TXA_2_, two similarly opposing vascular mediators, is also receptor-mediated.	**In vitro *studies:*** IP and TPα co-expression maintained their typical signalling (IP → cAMP; TP → inositol phosphates). ↑ cAMP production by TP activation in IP/TPα-expressing cells, an effect that was missing if either receptor is expressed alone or IP is absent. TP’s ability to trigger a PGI_2_-like cAMP response is enabled by IP/TPα dimerization, showcasing a new way IP can oppose TP signalling.	[54]
		Human genetic study with in vitro functional validation	A case–control study was performed to compare patients with coronary artery disease and those with non-synonymous mutations that reduced cAMP production (n = 23) with patients with non-synonymous mutations that had no reduction in cAMP (n = 17).	***Human studies:*** Through human population-based studies (n = 1761), 18 non-synonymous mutations were discovered. **In vitro *studies:*** Mutations (M113T, L104R, and R279C) in three highly conserved positions showed severe misfolding that caused impaired binding and activation of cell surface receptors. Normal signalling (via cAMP) of hIP agonist played a key cardioprotective role.	[55]

COS-1: CV-1 in Origin, Simian, SV40-transformed; hIP: human prostacyclin receptor; cAMP: cyclic adenosine monophosphate; IP: prostacyclin receptor; PGI2: prostacyclin; TP: thromboxane prostanoid receptor; TXA_2_: thromboxane A_2_; TPα: thromboxane A2 receptor alpha isoform.

**Table 3:** TP receptor modulation and the related endothelial inflammation. ↑: Increase; ↓: decrease.

Authors, Year	Review	Types of Studies Included in Review	Aim of Studies	Summary of Results	Ref.
Félétou M, et al. 2010 [37]	(TP receptors and oxidative stress hand in hand from endothelial dysfunction to atherosclerosis)	In vitro and in vivo studies	To assess the effect of S18886 on reducing atherogenesis in diabetic apoE−/− mice.	**In vivo *studies:*** ↓ S18886 significantly prevented a diabetes-related increase in lesion area. ↓ S18886 significantly decreased endothelial dysfunction. **In vitro *studies:*** ↓ Endothelial nitric oxide synthase expression.	[38]
		In vivo study	To assess how TXA 2 and PGI 2 contribute to the formation of atherosclerotic lesions in mice without prostanoid receptors via homologous recombination.	**In vivo *studies:*** In apoE–/–IP–/– mice, plaques displayed a partial endothelial rupture. ↑ Expression of ICAM-1. ↓ Expression of PECAM-1. Compared to apoE−/− mice (apoE−/−), ex vivo platelet activation with thrombin displayed increased and decreased surface P-selectin expression sensitivity in apoE−/−IP−/− and apoE−/−TP−/− mice, respectively.	[39]
		In vitro study	To assess how U46619, a TPr agonist, affects IL-1β’s VCAM-1 expression in VSMCs.	**In vitro *studies:*** NF-κB activation is unaffected by U46619 alone. VCAM-1 expression is not induced by U46619. Through the JNK signalling pathway, U46619 increases VCAM-1 expression, causing an increase in AP-1 protein activity and VCAM-1 gene transcription.	[40]

TXA2: thromboxane A_2_; PGI2: prostacyclin; ICAM-1: Intercellular Adhesion Molecule 1; PECAM-1: Platelet Endothelial Cell Adhesion Molecule-1; NF-κB: nuclear factor-Κb; JNK: c-jun N-terminal kinase; VSMCs: vascular smooth muscle cells; AP-1: Activator Protein 1.

**Table 4:** The role of prostanoids in vascular remodelling and platelet dysfunction. ↑: Increase; ↓: decrease.

Authors, Year	Review	Types of Studies Included in Review	Aim of Studies	Summary of Results	Ref.
Yuhki K, et al. 2011 [42]	(Roles of prostanoids in the pathogenesis of cardiovascular diseases: Novel insights from knockout mouse studies)	In vitro and in vivo studies	To assess if PGI2 influences EPCs to control vascular remodelling.	**In vitro *studies:*** ↓ Adhesion, migration, and proliferation of EPCs on fibronectin in IP-deficient EPCs compared to WT EPCs. **In vivo *studies:*** WT EPCs, but not IP-deficient EPCs, contributed to reendothelialization and successfully reversed accelerated vascular remodelling.	[43]
		In vitro and ex vivo studies	To examine the impact of PGE_2_ and CL 115,347, its antihypertensive counterpart, on human platelet function and vascular contractility in vitro.	**Ex vivo *studies:*** ↑ ADP- and collagen-induced aggregation at low concentrations by PGE_2_. ↓ PGE_2_ inhibiting it at high concentrations. **In vitro *studies:*** CL 115,347 showed a small impact at low levels but amplified aggregation at high levels. CL 115,347 exhibited a reduced activity on platelet activity and vascular contractility after in vitro modulation with respect to PGE_2_.	[29]
		In vitro and in vivo studies	To investigate how PGE2 regulates platelet function, affecting haemostasis and thromboembolism, using EP3−/− mice.	**In vitro *studies:*** PGE2 amplified platelet aggregation caused by U46619 through EP3 by increasing [Ca^2+^]i, lowering [cAMP]i, or a combination of both. **In vivo *studies:*** EP3−/− mice showed considerably extended bleeding times in vivo.	[28]

PGI2: prostacyclin; EPCs: endothelial progenitor cells; ADP: adenosine diphosphate; PGE2: prostaglandin E2; EP3: prostaglandin E2 receptor 3; cAMP: cyclic adenosine monophosphate.

**Table 5:** The genetic and molecular mechanisms underlying atherosclerotic plaque instability. ↑: Increase; ↓: decrease.

Authors, Year	Review	Types of Studies Included in Review	Aim of Studies	Summary of Results	Ref.
Cipollone F, et al. 2010 [45]	**(Genetic and molecular determinants of atherosclerotic plaque instability)**	Observational study with in vitro analysis	To analyse EP1–4 expression in plaques from patients (symptomatic and asymptomatic), and relate it to inflammatory infiltration, COX-2/mPGES-1 and MMP expression, and patient clinical features.	***Human studies:*** MMP-rich symptomatic lesions had a greater amount of EP4. The over-expression of EP4 correlated with increased inflammation in atherosclerotic plaques. Symptomatic and asymptomatic plaques showed no difference in EP2. **In vitro *studies:*** The EP4 antagonist L-161 982 suppressed MMP induction by PGE2.	[46]
		Observational clinical study on human tissue	To determine the link between COX-2/PGES expression in carotid plaques, inflammatory infiltration/MMP activity, and clinical patient presentation.	***Human studies:*** Symptomatic plaques had a significantly higher percentage of macrophage-rich areas (*p* < 0.0001). Activated MMPs were found in all symptomatic plaques. **In vitro *studies:*** ↓ COX-2 by NS-398 also lowered MMP production; PGE2 reversed this.	[59]
		Observational clinical study on human tissue	To connect MMP properties, levels, and expression in carotid plaques with clinical condition, embolization of the brain, and tissue analysis.	***Human studies:*** The MMP-9 level was 125.7 ng/mL (median) in group 4 and below 32 ng/mL (median) in all other groups (*p* = 0.003). No difference was observed in MMPs 1, 2, or 3 levels. ↑ MMP-9 in plaques with spontaneous embolization (*p* = 0.019) and histological instability (*p* < 0.03).	[31]

EP1: prostaglandin E2 receptor 1; MMP: metalloproteinases; EP2: prostaglandin E2 receptor 2; EP4: prostaglandin E2 receptor 4; PGE2: prostaglandin E2; COX-2/mPGES-1: cyclooxygenase 2/microsomal prostaglandin E synthase-1; COX-2: cyclooxygenase 2; MMP-9: metalloproteinases 9.

## Data Availability

No new data were created or analysed in this study. Data sharing is not applicable to this article.

## Abbreviations

12-HETE	12-hydroxyeicosatetraenoic acid
15-HETE	15-hydroxyeicosatetraenoic acid
5-HETE	5-hydroxyeicosatetraenoic Acid
AA	arachidonic acid
ADP	adenosine diphosphate
AP-1	Activator Protein 1
aPC	activated protein C
Apo-E	apolipoprotein E
cGMP	cyclic guanosine monophosphate
COS-1	CV-1 in Origin, Simian, SV40-transformed
cAMP	cyclic adenosine monophosphate
COX	cyclooxygenase
COX-1	cyclooxygenase 1
COX-2	cyclooxygenase 2
COX-2/mPGES-1	cyclooxygenase 2/microsomal prostaglandin E synthase-1
cPLA2	cytosolic phospholipase A2
DP1	D-type prostanoid receptor 1
DP2	D-type prostanoid receptor 2
DVT	deep vein thrombosis
ECM	extracellular matrix
ECs	endothelial cells
EET	epoxycosatrienoic acids
eNOS	endothelial nitric oxide synthase
EP	E-type prostanoid receptors
EP1	prostaglandin E2 receptor 1
EP2	prostaglandin E2 receptor 2
EP3	prostaglandin E2 receptor 3
EP3α	prostaglandin E2 receptor 3 alpha
EP3β	prostaglandin E2 receptor 3 beta
EP4	prostaglandin E2 receptor 4
EPCs	endothelial progenitor cells
ER	endoplasmic reticulum
ET-1	endothelin-1
F_2_α	prostaglandin F2α
FcεRI	high-affinity IgE receptor
FP	prostaglandin F receptor
GPCRs	G protein-coupled receptors
GWASs	genome-wide association studies
HDAC	Histone Deacetylase
HETE	hydroxycosatetraenoic acids
hIP	human prostacyclin receptor
IBD	Inflammatory Bowel Disease
ICAM-1	Intercellular Adhesion Molecule 1
IL-1β	interleukin-1 beta
IL-4	Interleukin-4
IP	prostacyclin receptor
JNK	c-jun N-terminal kinase
KIT	proto-oncogene, receptor tyrosine kinase
LDL	low-density lipoprotein
LOX	lipoxygenase
LPS	Lipopolysaccharide
LTB_4_	leukotriene B4
LTC_4_	leukotriene C4
LTD_4_	leukotriene D4
LTE_4_	leukotriene E4
MMP	metalloproteinases
MMP-2	metalloproteinases 2
MMP-9	metalloproteinases 9
MMPs	Matrix Metalloproteinases
mPGES-1	microsomal prostaglandin E synthase-1
mRNA	messenger ribonucleic acid
NF-κB	nuclear factor-κB
NO	nitric oxide
NSAIDs	non-steroidal anti-inflammatory drugs
ox-LDL	oxidized low-density lipoprotein
PECAM-1	Platelet Endothelial Cell Adhesion Molecule-1
PGD_2_	prostaglandin D2
PGE	prostaglandin E
PGE_2_	prostaglandin E2
PGG_2_	prostaglandin G2
PGH_2_	prostaglandin H2
PGHS	prostaglandin H synthase
PGI	prostaglandin I
PGI_2_	prostacyclin
PI3K/Akt	phosphatidylinositol 3-kinase/protein kinase B
PICO	Population Intervention Comparison Outcome Population
PRISMA-ScR	Preferred Reporting Items for Scoping Reviews and Meta-Analyses extension for Scoping Reviews
PTGIR	Prostaglandin I2 Receptor gene polymorphisms
ROS	reactive oxygen species
SCF	Stem Cell Factor
SCFAs	Short-Chain Fatty Acids
SMCs	smooth muscle cells
TF	Tissue Factor
TFPI	the tissue factor pathway inhibitor
TM	thrombomodulin
TP	thromboxane prostanoid receptor
TPα	thromboxane A2 receptor alpha isoform
TXA_2_	thromboxane A_2_
TXs	thromboxanes
VCAM1	vascular cell adhesion molecule 1
VSMCs	vascular smooth muscle cells

## References

[1] Zamanian R.T., Kudelko K.T., Sung Y.K., Perez V.J., Liu J., Spiekerkoetter E. (2014). Current clinical management of pulmonary arterial hypertension. Circ. Res..

[2] Krüger-Genge A., Blocki A., Franke R.-P., Jung F. (2019). Vascular Endothelial Cell Biology: An Update. Int. J. Mol. Sci..

[3] Gimbrone M.A., García-Cardeña G. (2016). Endothelial Cell Dysfunction and the Pathobiology of Atherosclerosis. Circ. Res..

[4] Gimbrone M.A., García-Cardeña G. (2013). Vascular endothelium, hemodynamics, and the pathobiology of atherosclerosis. Cardiovasc. Pathol..

[5] Neubauer K., Zieger B. (2022). Endothelial cells and coagulation. Cell Tissue Res..

[6] Medina-Leyte D.J., Zepeda-García O., Domínguez-Pérez M., González-Garrido A., Villarreal-Molina T., Jacobo-Albavera L. (2021). Endothelial Dysfunction, Inflammation and Coronary Artery Disease: Potential Biomarkers and Promising Therapeutical Approaches. Int. J. Mol. Sci..

[7] Peng Z., Shu B., Zhang Y., Wang M. (2019). Endothelial Response to Pathophysiological Stress. Arter. Thromb. Vasc. Biol..

[8] Nguyen A.B., Iqbal O., Block R.C., Mousa S.A. (2023). Prevention and treatment of atherothrombosis: Potential impact of nanotechnology. Vasc. Pharmacol..

[9] Mallick R., Duttaroy A.K. (2022). Modulation of endothelium function by fatty acids. Mol. Cell. Biochem..

[10] Bochenek M.L., Schäfer K. (2019). Role of Endothelial Cells in Acute and Chronic Thrombosis. Hamostaseologie.

[11] Immanuel J., Yun S. (2023). Vascular Inflammatory Diseases and Endothelial Phenotypes. Cells.

[12] Naderi-Meshkin H., Setyaningsih W.A.W. (2024). Endothelial Cell Dysfunction: Onset, Progression, and Consequences. Front. Biosci..

[13] Libby P., Buring J.E., Badimon L., Hansson G.K., Deanfield J., Bittencourt M.S., Tokgözoğlu L., Lewis E.F. (2019). Atherosclerosis. Nat. Rev. Dis. Primers.

[14] Daiber A., Chlopicki S. (2020). Revisiting pharmacology of oxidative stress and endothelial dysfunction in cardiovascular disease: Evidence for redox-based therapies. Free Radic. Biol. Med..

[15] Dabravolski S., Orekhov N.A., Melnichenko A., Sukhorukov V.N., Popov M.A., Orekhov A. (2024). Cholesteryl Ester Transfer Protein (CETP) Variations in Relation to Lipid Profiles and Cardiovascular Diseases: An Update. Curr. Pharm. Des..

[16] Wang R., Wang M., Ye J., Sun G., Sun X. (2021). Mechanism overview and target mining of atherosclerosis: Endothelial cell injury in atherosclerosis is regulated by glycolysis (Review). Int. J. Mol. Med..

[17] Asada Y., Yamashita A., Sato Y., Hatakeyama K. (2020). Pathophysiology of atherothrombosis: Mechanisms of thrombus formation on disrupted atherosclerotic plaques. Pathol. Int..

[18] Ohman E.M., Bhatt D.L., Steg P.G., Goto S., Hirsch A.T., Liau C.S., Mas J.L., Richard A.J., Röther J., Wilson P.W. (2006). The REduction of Atherothrombosis for Continued Health (REACH) Registry: An international prospective observational investigation in subjects at risk for atherothrombotic events-study design. Am. Heart J..

[19] Yamashita A., Asada Y. (2023). Underlying mechanisms of thrombus formation/growth in atherothrombosis and deep vein thrombosis. Pathol. Int..

[20] Moreno J.J. (2017). Eicosanoid receptors: Targets for the treatment of disrupted intestinal epithelial homeostasis. Eur. J. Pharmacol..

[21] Biringer R.G. (2022). A review of non-prostanoid, eicosanoid receptors: Expression, characterization, regulation, and mechanism of action. J. Cell Commun. Signal..

[22] Piper K., Garelnabi M. (2020). Eicosanoids: Atherosclerosis and cardiometabolic health. J. Clin. Transl. Endocrinol..

[23] Calder P.C. (2020). Eicosanoids. Essays Biochem..

[24] Wang B., Wu L., Chen J., Dong L., Chen C., Wen Z., Hu J., Fleming I., Wang D.W. (2021). Metabolism pathways of arachidonic acids: Mechanisms and potential therapeutic targets. Signal Transduct. Target. Ther..

[25] Zhou Y., Khan H., Xiao J., Cheang W.S. (2021). Effects of Arachidonic Acid Metabolites on Cardiovascular Health and Disease. Int. J. Mol. Sci..

[26] Patrignani P., Di Febbo C., Tacconelli S., Douville K., Guglielmi M.D., Horvath R.J., Ding M., Sierra K., Stitham J., Gleim S. (2008). Differential association between human prostacyclin receptor polymorphisms and the development of venous thrombosis and intimal hyperplasia: A clinical biomarker study. Pharmacogenet. Genom..

[27] Gross S., Tilly P., Hentsch D., Vonesch J.L., Fabre J.E. (2007). Vascular wall-produced prostaglandin E2 exacerbates arterial thrombosis and atherothrombosis through platelet EP3 receptors. J. Exp. Med..

[28] Ma H., Hara A., Xiao C.Y., Okada Y., Takahata O., Nakaya K., Sugimoto Y., Ichikawa A., Narumiya S., Ushikubi F. (2001). Increased bleeding tendency and decreased susceptibility to thromboembolism in mice lacking the prostaglandin E receptor subtype EP_3_. Circulation.

[29] Gray S.J., Heptinstall S. (1985). The effects of PGE2 and CL 115,347, an antihypertensive PGE2 analogue, on human blood platelet behaviour and vascular contractility. Eur. J. Pharmacol..

[30] Babaev V.R., Chew J.D., Ding L., Davis S., Breyer M.D., Breyer R.M., Oates J.A., Fazio S., Linton M.F. (2008). Macrophage EP4 deficiency increases apoptosis and suppresses early atherosclerosis. Cell Metab..

[31] Loftus I.M., Naylor A.R., Goodall S., Crowther M., Jones L., Bell P.R., Thompson M.M. (2000). Increased matrix metalloproteinase-9 activity in unstable carotid plaques. A potential role in acute plaque disruption. Stroke.

[32] Pavlovic S., Du B., Sakamoto K., Khan K.M., Natarajan C., Breyer R.M., Dannenberg A.J., Falcone D.J. (2006). Targeting prostaglandin E2 receptors as an alternative strategy to block cyclooxygenase-2-dependent extracellular matrix-induced matrix metalloproteinase-9 expression by macrophages. J. Biol. Chem..

[33] Yang C., Liu X., Cao Q., Liang Q., Qiu X. (2011). Prostaglandin E receptors as inflammatory therapeutic targets for atherosclerosis. Life Sci..

[34] Imig J.D. (2020). Eicosanoid blood vessel regulation in physiological and pathological states. Clin. Sci..

[35] Tricco A.C., Lillie E., Zarin W., O’Brien K.K., Colquhoun H., Levac D., Moher D., Peters M.D.J., Horsley T., Weeks L. (2018). PRISMA Extension for Scoping Reviews (PRISMA-ScR): Checklist and Explanation. Ann. Intern. Med..

[36] Gleim S., Stitham J., Tang W.H., Martin K.A., Hwa J. (2012). An eicosanoid-centric view of atherothrombotic risk factors. Cell. Mol. Life Sci..

[37] Félétou M., Cohen R.A., Vanhoutte P.M., Verbeuren T.J. (2010). TP receptors and oxidative stress hand in hand from endothelial dysfunction to atherosclerosis. Adv. Pharmacol..

[38] Zuccollo A., Shi C., Mastroianni R., Maitland-Toolan K.A., Weisbrod R.M., Zang M., Xu S., Jiang B., Oliver-Krasinski J.M., Cayatte A.J. (2005). The thromboxane A_2_ receptor antagonist S18886 prevents enhanced atherogenesis caused by diabetes mellitus. Circulation.

[39] Kobayashi T., Tahara Y., Matsumoto M., Iguchi M., Sano H., Murayama T., Arai H., Oida H., Yurugi-Kobayashi T., Yamashita J.K. (2004). Roles of thromboxane A_2_ and prostacyclin in the development of atherosclerosis in apoE-deficient mice. J. Clin. Investig..

[40] Bayat H., Xu S., Pimentel D., Cohen R.A., Jiang B. (2008). Activation of thromboxane receptor upregulates interleukin (IL)-1β-induced VCAM-1 expression through JNK signaling. Arter. Thromb. Vasc. Biol..

[41] Félétou M., Vanhoutte P.M. (2006). Endothelial dysfunction: A multifaceted disorder (The Wiggers Award Lecture). Am. J. Physiol. Heart Circ. Physiol..

[42] Yuhki K., Kojima F., Kashiwagi H., Kawabe J., Fujino T., Narumiya S., Ushikubi F. (2011). Roles of prostanoids in the pathogenesis of cardiovascular diseases: Novel insights from knockout mouse studies. Pharmacol. Ther..

[43] Kawabe J., Yuhki K., Okada M., Kanno T., Yamauchi A., Tashiro N., Sasaki T., Okumura S., Nakagawa N., Aburakawa Y. (2010). Prostaglandin I_2_ promotes recruitment of endothelial progenitor cells and limits vascular remodeling. Arter. Thromb. Vasc. Biol..

[44] He T., Lu T., d’Uscio L.V., Lam C.F., Lee H.C., Katusic Z.S. (2008). Angiogenic function of prostacyclin biosynthesis in human endothelial progenitor cells. Circ. Res..

[45] Cipollone F., Toniato E., Martinotti S., Mezzetti A. (2010). Genetic and molecular determinants of atherosclerotic plaque instability. Curr. Vasc. Pharmacol..

[46] Cipollone F., Fazia M.L., Iezzi A., Cuccurullo C., De Cesare D., Ucchino S., Spigonardo F., Marchetti A., Buttitta F., Paloscia L. (2005). Association between prostaglandin E receptor subtype EP4 overexpression and unstable phenotype in atherosclerotic plaques in human. Arter. Thromb. Vasc. Biol..

[47] Faour W.H., He Y., He Q.W., de Ladurantaye M., Quintero M., Mancini A., Di Battista J.A. (2001). Prostaglandin E_2_ regulates the level and stability of cyclooxygenase-2 mRNA through activation of p38 mitogen-activated protein kinase in interleukin-1 beta-treated human synovial fibroblasts. J. Biol. Chem..

[48] Rabausch K., Bretschneider E., Sarbia M., Meyer-Kirchrath J., Censarek P., Pape R., Fischer J.W., Schrör K., Weber A.A. (2005). Regulation of thrombomodulin expression in human vascular smooth muscle cells by COX-2-derived prostaglandins. Circ. Res..

[49] Meyer-Kirchrath J., Debey S., Glandorff C., Kirchrath L., Schrör K. (2004). Gene expression profile of the Gs-coupled prostacyclin receptor in human vascular smooth muscle cells. Biochem. Pharmacol..

[50] Hong T.T., Huang J., Barrett T.D., Lucchesi B.R. (2008). Effects of cyclooxygenase inhibition on canine coronary artery blood flow and thrombosis. Am. J. Physiol. Heart Circ. Physiol..

[51] Crofford L.J., Oates J.C., McCune W.J., Gupta S., Kaplan M.J., Catella-Lawson F., Morrow J.D., McDonagh K.T., Schmaier A.H. (2000). Thrombosis in patients with connective tissue diseases treated with specific cyclooxygenase 2 inhibitors: A report of four cases. Arthritis Rheum..

[52] Arehart E., Stitham J., Asselbergs F.W., Douville K., MacKenzie T., Fetalvero K.M., Gleim S., Kasza Z., Rao Y., Martel L. (2008). Acceleration of cardiovascular disease by a dysfunctional prostacyclin receptor mutation: Potential implications for cyclooxygenase-2 inhibition. Circ. Res..

[53] Ibrahim S., Tetruashvily M., Frey A.J., Wilson S.J., Stitham J., Hwa J., Smyth E.M. (2010). Dominant negative actions of human prostacyclin receptor variant through dimerization: Implications for cardiovascular disease. Arter. Thromb. Vasc. Biol..

[54] Wilson S.J., Roche A.M., Kostetskaia E., Smyth E.M. (2004). Dimerization of the human receptors for prostacyclin and thromboxane facilitates thromboxane receptor-mediated cAMP generation. J. Biol. Chem..

[55] Stitham J., Arehart E., Elderon L., Gleim S.R., Douville K., Kasza Z., Fetalvero K., MacKenzie T., Robb J., Martin K.A. (2011). Comprehensive biochemical analysis of rare prostacyclin receptor variants: Study of association of signaling with coronary artery obstruction. J. Biol. Chem..

[56] Irie A., Sugimoto Y., Namba T., Harazono A., Honda A., Watabe A., Negishi M., Narumiya S., Ichikawa A. (1993). Third isoform of the prostaglandin-E-receptor EP3 subtype with different C-terminal tail coupling to both stimulation and inhibition of adenylate cyclase. Eur. J. Biochem..

[57] Galis Z.S., Muszynski M., Sukhova G.K., Simon-Morrissey E., Unemori E.N., Lark M.W., Amento E., Libby P. (1994). Cytokine-stimulated human vascular smooth muscle cells synthesize a complement of enzymes required for extracellular matrix digestion. Circ. Res..

[58] Newby A.C. (2007). Metalloproteinases and vulnerable atherosclerotic plaques. Trends Cardiovasc. Med..

[59] Cipollone F., Prontera C., Pini B., Marini M., Fazia M., De Cesare D., Iezzi A., Ucchino S., Boccoli G., Saba V. (2001). Overexpression of functionally coupled cyclooxygenase-2 and prostaglandin E synthase in symptomatic atherosclerotic plaques as a basis of prostaglandin E_2_-dependent plaque instability. Circulation.

[60] Badimon L., Peña E., Arderiu G., Padró T., Slevin M., Vilahur G., Chiva-Blanch G. (2018). C-Reactive Protein in Atherothrombosis and Angiogenesis. Front. Immunol..

[61] Baigent C., Collins R., Appleby P., Parish S., Sleight P., Peto R. (1998). ISIS-2: 10 year survival among patients with suspected acute myocardial infarction in randomised comparison of intravenous streptokinase, oral aspirin, both, or neither. The ISIS-2 (Second International Study of Infarct Survival) Collaborative Group. BMJ.

[62] Kurata N., Tokashiki N., Fukushima K., Misao T., Hasuoka N., Kitagawa K., Mashimo M., Regan J.W., Murayama T., Fujino H. (2019). Short chain fatty acid butyrate uptake reduces expressions of prostanoid EP_4_ receptors and their mediation of cyclooxygenase-2 induction in HCA-7 human colon cancer cells. Eur. J. Pharmacol..

[63] Mao Y.-H., Song F.-L., Xu Y.-X., Song A.-X., Wang Z.-M., Zhao M.-Z., He F., Tian Z.-Z., Yang Y. (2022). Extraction, Characterization, and Platelet Inhibitory Effects of Two Polysaccharides from the Cs-4 Fungus. Int. J. Mol. Sci..

[64] Jing L., Yanyan Z., Junfeng F. (2015). Acetic Acid in Aged Vinegar Affects Molecular Targets for Thrombus Disease Management. Food Funct..

[65] Takachi R., Kimira M., Uesugi S., Kudo Y., Ouchi K., Watanabe S. (2004). The effect of dietary and plasma fatty acids on platelet aggregation in senior generation of Japanese women. Biofactors.

[66] Fujino H. (2025). Butyrate, IL-4, and EP4 Receptors: A Triad of Colorectal Homeostasis, Protecting Against Onset of Cancer and IBD?. Bioessays.

[67] Salvi P.S., Cowles R.A. (2021). Butyrate and the Intestinal Epithelium: Modulation of Proliferation and Inflammation in Homeostasis and Disease. Cells.

[68] Kunikata T., Yamane H., Segi E., Matsuoka T., Sugimoto Y., Tanaka S., Tanaka H., Nagai H., Ichikawa A., Narumiya S. (2005). Suppression of allergic inflammation by the prostaglandin E receptor subtype EP3. Nat. Immunol..

[69] Ohira H., Tsutsui W., Mamoto R., Yamaguchi S., Nishida M., Ito M., Fujioka Y. (2016). Butyrate attenuates lipolysis in adipocytes co-cultured with macrophages through non-prostaglandin E2-mediated and prostaglandin E2-mediated pathways. Lipids Health Dis..

[70] Gudneppanavar R., Sabu Kattuman E.E., Teegala L.R., Southard E., Tummala R., Joe B., Thodeti C.K., Paruchuri S. (2023). Epigenetic histone modification by butyrate downregulates KIT and attenuates mast cell function. J. Cell. Mol. Med..

